# Influence of Material Properties on the Damage-Reporting and Self-Healing Performance of a Mechanically Active Dynamic Network Polymer in Coating Applications

**DOI:** 10.3390/molecules26092468

**Published:** 2021-04-23

**Authors:** Da Hae Son, Gi Young Kim, Ji-Eun Jeong, Sang-Ho Lee, Young Il Park, Hoyoul Kong, In Woo Cheong, Jin Chul Kim

**Affiliations:** 1 Center for Advanced Specialty Chemicals, Division of Specialty and Bio-based Chemicals Technology, Korea Research Institute of Chemical Technology (KRICT), Ulsan 44412, Korea; sdf1114@krict.re.kr (D.H.S.); giyoung@krict.re.kr (G.Y.K.); jieunj@krict.re.kr (J.-E.J.); slee@krict.re.kr (S.-H.L.); ypark@krict.re.kr (Y.I.P.); hkong2@krict.re.kr (H.K.); 2 School of Applied Chemistry, Kyungpook National University, Daegu 41566, Korea

**Keywords:** polymer coating, dynamic polymer network, mechanophore, self-healing coating, self-reporting polymer

## Abstract

We conducted a detailed investigation of the influence of the material properties of dynamic polymer network coatings on their self-healing and damage-reporting performance. A series of reversible polyacrylate urethane networks containing the damage-reporting diarylbibenzofuranone unit were synthesized, and their material properties (e.g., indentation modulus, hardness modulus, and glass-transition temperature) were measured conducting nanoindentation and differential scanning calorimetry experiments. The damage-reporting and self-healing performances of the dynamic polymer network coatings exhibited opposite tendencies with respect to the material properties of the polymer network coatings. Soft polymer network coatings with low glass-transition temperature (~10 °C) and indentation hardness (20 MPa) exhibited better self-healing performance (almost 100%) but two times worse damage-reporting properties than hard polymer network coatings with high glass-transition temperature (35~50 °C) and indentation hardness (150~200 MPa). These features of the dynamic polymer network coatings are unique; they are not observed in elastomers, films, and hydrogels, whereby the polymer networks are bound to the substrate surface. Evidence indicates that controlling the polymer’s physical properties is a key factor in designing high-performance self-healing and damage-reporting polymer coatings based on mechanophores.

## 1. Introduction

Mechanophores are molecules that can undergo chemical changes in response to an externally applied mechanical force [1,2,3,4,5,6]. Notably, some mechanophores are also chromophores, meaning that they can both undergo chemical changes and alter their light-emitting characteristics in response to a mechanical stimulus [7,8]. Chemical motifs that are able to behave as both mechanophores and chromophores have been embedded into polymers to fabricate systems that could function as damage-reporting materials, such as stress sensors and visual stress–strain detectors. Recently, extensive efforts have been devoted to using these light-emitting polymers as self-healing materials that are able to signal their damaged or healed status [9,10,11]. Thus far, however, most of these investigations have focused exclusively on films and elastomers, although the combination of these technologies (self-healing and damage-reporting technologies) is also important in the development of novel self-healing polymer coatings.

Self-healing coatings can be classified into two categories: extrinsic and intrinsic. Extrinsic self-healing coatings are obtained by including in the said coatings microcapsules with the ability to heal the damaged area [12]. In the case of these coatings, the damage-reporting function can be easily achieved by embedding damage indicators, such as pH-sensitive dyes and fluorescent molecules, into the microcapsules comprising healing agents [13,14,15]. In this system, the material properties and forms of matrix polymers rarely influence self-healing and damage-reporting performances, since self-healing and damage-reporting are elicited by the diffusion of the solutions of the healing agents and/or the damage indicators. However, the extrinsic self-healing mechanism limits repeated use of self-healing and damage-reporting functions because the said mechanism depends on the collapse of the microcapsules.

**Figure 1:**
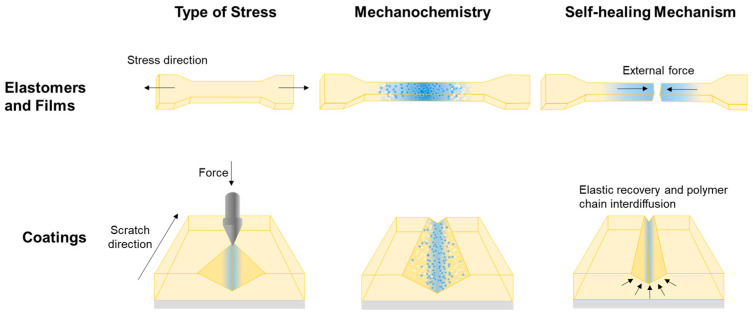
Differences in the self-healing mechanisms of intrinsic self-healing elastomers, films, and coatings.

In the case of intrinsic self-healing coatings based on reversible processes, the self-healing process differs completely from those occurring with other forms of polymer network materials, such as elastomers, adhesives, and gels, because the polymer networks are bound to the substrate surface (Figure 1). Since the diffusion of polymer chains in the coatings is much more restricted than the diffusion of other polymer forms, elastic coatings with a low elastic modulus (*G′*) value are preferred over hard coatings to achieve high self-healing efficiency [16,17,18,19,20]. However, the effect of the material properties on the damage-reporting performance of a mechanically active dynamic network self-healing polymer coating has not yet been systematically reported. Recently, Geitner et al. reported a detailed investigation conducted on an intrinsic self-healing polymer coating using molecular coherent anti-Stokes Raman scattering analysis; notably, this investigation focused on the morphological and chemical aspects, and it did not deal with the effect that material properties have on damage-reporting and self-healing coatings [21].

In the present paper, we report a detailed investigation of the influence that the material properties of dynamic polymer network coatings have on their self-healing and damage-reporting performance. As a mechanophore, diarylbibenzofuranone (DABBF), first introduced by Hideyuki Otsuka’s group, was employed because this compound’s disintegration (triggered by mechanical activation) and recombination (proceeding via arylbenzofuranone radical coupling) can be visualized by a color change [22,23,24]. In addition, since the radical species generated as a result of the cleavage of a DABBF unit is known to tolerate the presence of oxygen and to be stable for prolonged periods of time under ambient conditions, the self-healing and damage-reporting performance of the dynamic polymer network can be reliably analyzed. A series of DABBF-containing poly(methyl methacrylate)-*co*-[poly(hydroxyethyl methacrylate)-*graft*-poly(oligo-caprolactone)] urethane networks exhibiting different material properties and different oligo-caprolactone bristle lengths of the polymer networks were prepared. The material properties (e.g., thermal transition temperature, thermal stability, and indentation hardness) were characterized by differential scanning calorimetry (DSC), thermogravimetric analysis (TGA), and nanoindentation testing. The surface damage-reporting and self-healing performance of the polymer networks were characterized using a micro-scratch tester equipped with an optical microscope. Finally, with these measurement data in hand, the relationship between the material properties and the damage-reporting and self-healing performance of the dynamic network polymers was systematically investigated.

## 2. Experiment

### 2.1. Materials

2,4-Di-*tert*-butylphenol (99%), acetic acid (≥99%), methanesulfonic acid (≥99%), tin(II) 2-ethylhexanoate (≥92.5%), ε-caprolactone (97%), toluene (≥99.5%), benzene (anhydrous, ≥99.8%), 2-butanone (99.7%), 2-hydroxyethyl methacrylate (HEMA, 97%), methyl methacrylate (MMA, 99%), and dibutyltin dilaurate (DBTDL, 95%) were purchased from Merck (Darmstadt, Germany). 3-chloro-1-propanol (>98%), di-*tert*-butyl peroxide (>98%), and 4-hydroxymandelic acid monohydrate were obtained from TCI (Tokyo, Japan). Azobisisobutyronitrile (AIBN, 98%) and sodium hydroxide (97%) were purchased from Junsei (Tokyo, Japan). For ring-opening polymerization, ε-caprolactone and toluene were distilled before use. MMA was passed through a basic alumina column to remove inhibitors before conducting the polymerization reaction. AIBN was recrystallized twice from methanol prior to use.

### 2.2. Synthesis

Synthesis of DABBF [22]. In a 100 mL two-neck round-bottom flask equipped with a reflux condenser, 2,4-di-*tert*-butylphenol (15 g, 73 mmol) and 4-hydroxymandelic acid monohydrate (10 g, 54 mmol) were dissolved in acetic acid (29 mL), and the resultant solution was stirred at 95 °C. Methanesulfonic acid (0.1 mL, 0.8 mmol) was then added to the acetic acid solution, and the reaction mixture thus obtained was stirred at 95 °C for 3 h. The mixture was then allowed to cool to room temperature, and it was made to stand overnight; the product that precipitated as a result was filtered and washed several times with water and hexane. After recrystallization from chloroform and hexane, precursor compound **1** was obtained as a white solid (yield = 56%). ^1^H-NMR (300 MHz, CDCl_3_) peak *δ* values (ppm): 7.31 (s, 1H), 7.09 (d, 2H), 7.04 (s, 1H), 6.82 (d, 2H), 4.94 (s, 1H), 4.77 (s, 1H), 1.43 (s, 9H), 1.29 (s, 9H).

Compound **1** (8 g, 24 mmol) was added to a 250 mL three-neck round-bottom flask equipped with a dropping funnel and reflux condenser; an aqueous sodium hydroxide solution (3 g NaOH in 60 mL of water) was then added to the flask. After the reaction mixture thus obtained was stirred at 80 °C under an Ar atmosphere, 3-chloro-1-propanol (4.2 mL, 50 mmol) was slowly dropped into the flask using the dropping funnel. The reaction mixture was maintained at 80 °C for 3 h. After the mixture was allowed to cool to room temperature, a solution of concentrated hydrochloric acid (7.6 mL in 60 mL water) was added to it, and the resulting mixture was kept at 80 °C for another 3 h. After the reaction mixture was allowed to cool to room temperature, the resultant precipitate was dissolved in ethyl ether, washed several times with brine, and then concentrated under reduced pressure. The crude product thus isolated was purified by column chromatography (using ethyl acetate:hexane = 3:7, *v*:*v*, as eluent). Precursor compound **2** was thus obtained as a white solid (yield = 64%). ^1^H-NMR (300 MHz, CDCl_3_) peak *δ* values (ppm): 7.31 (s, 1H), 7.16 (d, 2H), 7.04 (s, 1H), 6.92 (d, 2H), 4.78 (s, 1H), 4.12 (t, 2H), 3.86 (t, 2H), 2.05 (m, 2H), 1.43 (s, 9H), 1.29 (s, 9H).

Precursor **2** (5 g, 13 mmol), di-*tert*-butyl peroxide (19 g, 130 mmol), and benzene (25 mL) were added to a 100 mL round-bottom flask. The solution thus obtained was irradiated with UV light (365 nm) for 2 h at 30 °C using a photoreactor (LZC 4V, LUZCHEM, Canada). After completion of the reaction, the crude product was purified by flash column chromatography (using ethyl acetate:hexane = 3:1, *v*:*v*, as eluent) and recrystallized from a mixed-solvent system of chloroform and hexane to give DABBF as a white solid (yield = 40%). ^1^H-NMR (300 MHz, CDCl_3_) peak *δ* values (ppm): 7.29 (br, 8H), 6.82 (d, 4H), 4.14 (m, 4H), 3.88 (m, 4H), 2.06 (m, 4H), 1.31 (br, 18H), 1.17 (br, 18H).

Synthesis of oligo(ε-caprolactone) methacrylate monomers (MCLn). In a glovebox, ε-caprolactone, hydroxyethyl methacrylate, and tin(II) 2-ethylhexanoate were dissolved in toluene, and the solution thus obtained was stirred at 120 °C. After 2 h, excess methanol was added to the mixture to quench the reaction. The reaction mixture was precipitated in cold hexane, washed three times with cold hexane, and dried under vacuum at 30 °C for 12 h.

MCL1. Yield: 66%; ^1^H-NMR (300 MHz, CDCl_3_) peak *δ* values (ppm): 6.15 (s, 1H), 5.61 (s, 1H), 4.38 (s, 4H), 3.68 (t, 2H), 2.35 (m, 2H), 1.99 (s, 3H), 1.67 (m, 4H), 1.42 (m, 2H).

MCL5. Yield: 65%; ^1^H-NMR (300 MHz, CDCl_3_) peak *δ* values (ppm): 6.15 (s, 1H), 5.61 (s, 1H), 4.38 (s, 4H), 4.09 (t, 8H), 3.68 (t, 2H), 2.35 (m, 10H), 1.99 (s, 3H), 1.67 (m, 20H), 1.42 (m, 10H).

MCL10. Yield: 72%; ^1^H-NMR (300 MHz, CDCl_3_) peak *δ* values (ppm): 6.15 (s, 1H), 5.61 (s, 1H), 4.38 (s, 4H), 4.09 (t, 18H), 3.68 (t, 2H), 2.35 (m, 20H), 1.99 (s, 3H), 1.67 (br, 40H), 1.42 (br, 20H).

Polymerization of hydroxyl-terminated graft copolymers (GCLs). GCL1, GCL5, and GCL10 were synthesized starting from the corresponding MCLs (20 mol%) and methyl methacrylate (80 mol%) in 2-butanone via a conventional free-radical polymerization carried out in the presence of AIBN (2 mol%) at 75 °C for 3 h under Ar atmosphere. After being precipitated from diethyl ether, the polymer was washed and then dried under vacuum for 24 h.

GCL1. Yield: 70%; ^1^H-NMR (300 MHz, CDCl_3_) peak *δ* values (ppm): 4.30 (br, 4H), 3.61 (br, 14H), 2.37 (br, 2H), 1.68 (br, 4H), 1.42 (br, 2H).

GCL5. Yield: 58%; ^1^H-NMR (300 MHz, CDCl_3_) peak *δ* values (ppm): 4.30 (br, 4H), 4.19 (br, 8H), 3.61 (br, 14H), 2.37 (br, 10H), 1.68 (br, 20H), 1.42 (br, 10H).

GCL10. Yield: 45%; ^1^H-NMR (300 MHz, CDCl_3_) peak *δ* values (ppm): 4.30 (br, 4H), 4.19 (br, 18H), 3.61 (br, 14H), 2.37 (br, 20H), 1.68 (br, 40H), 1.42 (br, 20H).

### 2.3. Preparation of the Polymer Network Coatings (C-GCLs)

The GCL polymers, DABBF, Desmodur N3300, and a catalytic amount of DBTDL were dissolved in chloroform. The solution thus obtained was coated onto a glass slide implementing the conventional drawdown bar coating method. The coated glass slide was then cured in a convection oven at 70 °C for 12 h. The thickness of the coatings was adjusted to 40 μm. The weight contents of the DABBF units in the C-GCL coatings were adjusted to 22 wt%.

### 2.4. Chemical Structure Confirmation

^1^H-NMR spectra of the organic compounds and polymers were recorded using a 300 MHz NMR spectrometer (Bruker, Ultrashield, Fällanden, Switzerland) under ambient conditions. The singlet resonance peak due to residual CHCl_3_ in CDCl_3_ appearing at 7.26 ppm was selected as the reference standard.

### 2.5. Molecular-Weight Determination

The number-average molecular weight ($\bar{M_{n}}$) and dispersity (*Ð*_M_, = $\bar{M_{w}}$/$\bar{M_{n}}$, where $\bar{M_{w}}$ is the weight-average molecular weight) of the synthesized polymers were determined by size-exclusion chromatography using an apparatus (Agilent Tech 1260, Agilent Technologies, Santa Clara, CA, USA) equipped with a set of gel columns (Agilent PLgel 5 μm Mixed-D column, Agilent Technologies, Santa Clara, CA, USA). The system was equilibrated at 40 °C in anhydrous tetrahydrofuran (THF), which was used as both the polymer solvent and the eluent (flow rate of 1 mL min^−1^), and calibrated using polystyrene standards (650 ≤ $\bar{M_{w}}$ ≤ 6,375,000).

### 2.6. Thermal Property Determination

The thermal stability of the polymers was investigated by TGA (TA Instruments TGA Q500, New Castle, DE, USA) conducted under an N_2_ atmosphere between 25 and 600 °C applying a heating rate of 10 °C min^−1^. The thermal transitions of the polymers were determined from the second DSC heating ramp under a N_2_ atmosphere between −50 and 100 °C at a heating rate of 10 °C min^−1^ using a TA Instruments DSC Q2000.

### 2.7. Nanoindentation Test

Loading, holding, and unloading indentation measurements were performed using a nanoindentation tester (Anton Paar, NHT^3^, Graz, Austria) equipped with a Berkovich-type indenter. During the loading step, the force imparted by the indenter was gradually increased from 0 to 20 mN at a rate of 20 mN min^−1^. During the holding step, the applied force was maintained at a value of 20 mN for 30 s. Finally, the indenter was unloaded at the same absolute value of the rate used for the loading. Through these three steps, the values for the maximum displacement (*h*_max_), permanent depth of penetration (final depth, *h*_f_), elastic unloading stiffness (*S* = d*P*/d*h*), indentation hardness (*H*_IT_), and indentation modulus (*E*_IT_) were obtained [17].

### 2.8. Micro-Scratch Test

Micro-scratch *tests* were performed using a spheroconical indenter (tip radius: 5 μm) mounted on a scratch test machine (Anton Paar, nano-scratch tester). The force exerted on the coating was progressively increased from 2 to 100 mN to initiate fracture formation on the coating surface. The indenter speed and the scratch length were 2 mm min^−1^ and 2.0 mm, respectively. The mechanisms for the fracture of the polymer coatings and for the dissociation of the DABBF units present in the polymer networks (dissociation of the polymer networks) were analyzed using an optical microscope attached to the scratch test machine [17].

### 2.9. Scratch-Healing Efficiency Determination

Scratch healing was induced by heating the material at 60 °C for 6 h. The healing of the scratches and the recombination of the DABBF at the damaged surfaces were observed using an optical microscope attached to the scratch test machine.

### 2.10. Measurement of DABBF Dissociation and Recombination Efficiencies

The dissociation efficiency of DABBF caused by the generation of the surface scratch and the recombination efficiency of DABBF influenced by the self-healing process of the C-GCL coatings were quantitatively determined in terms of the changes in the value of Db^*^. In the CIELAB color space (L*a*b*), the b* axis represents the blue-yellow components, with number toward blue and positive toward yellow. The Db* transition measurement of the scratched and healed surface of the C-GCL coatings was conducted by analyzing the color coordination of pixels in the optical microscopy images. The CIE 1931 XYZ color space values of the pixels in the image were converted to the L*a*b* values using the Adobe Photoshop® program (version 12.0 x64). Notably, the Db* value at a certain point was the average value of 100 pixels.

## 3. Results and discussion

### 3.1. Material Design and Preparation

**Scheme 1:**
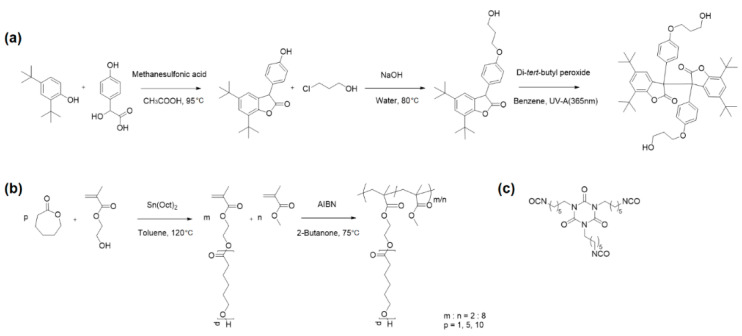
Procedures implemented to synthesize (**a**) diarylbibenzofuranone and (**b**) oligo(ε-caprolactone) methacrylate monomers and hydroxyl-terminated graft copolymers; (**c**) chemical structure of polyisocyanate (Desmodur N3300).

The detailed procedures for the syntheses of DABBF, MCLs, and GCLs are reported in Scheme 1.

**Figure 2:**
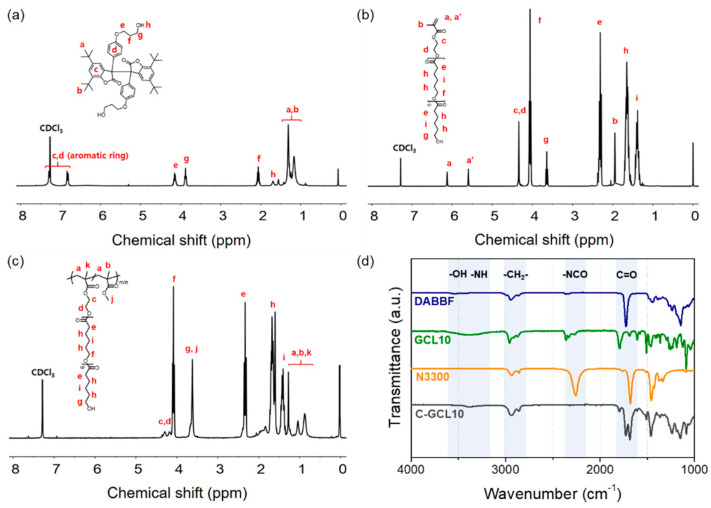
Chemical structure confirmation of the synthesized materials: ^1^H-NMR spectra of (**a**) diarylbibenzofuranone (DABBF), (**b**) oligo(ε-caprolactone) methacrylate 10 monomer, and (**c**) hydroxyl-terminated graft 10 polymer (GCL10). (**d**) Fourier-transform infrared spectra of DABBF, hydroxyl-terminated graft polymer (GCL5, polyisocyanate (Desmodur N3300)), and C-GCL10.

DABBF was prepared from 4-hydroxymandelic acid and 2,4-di-*tert*-butylphenol in three steps following a previously reported protocol. MCL1, MCL5, and MCL10 were synthesized via ring-opening polymerization of the ε-caprolactone monomer using the hydroxyethyl methacrylate monomer as initiator, in the presence of tin(II) 2-ethylhexanoate acting as a catalyst. GCL1, GCL5, and GCL10 were synthesized from MMA and the corresponding MCL monomers in the presence of AIBN acting as the initiator by way of free-radical polymerization. The chemical structures of the synthesized materials were confirmed by ^1^H-NMR spectroscopy (Figure 2). Values for the average molecular weights, degrees of polymerization, and monomer ratios of MCLs and GCL polymers are listed in Table 1 and Table 2.

**Figure 3:**
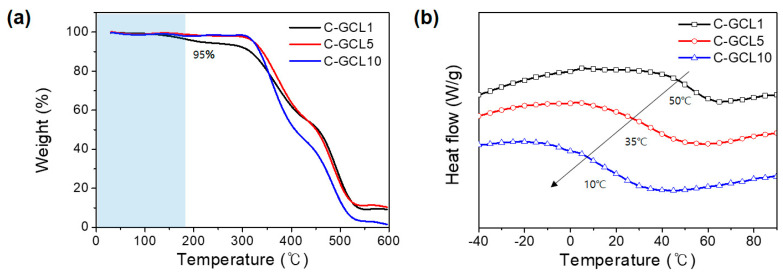
Thermal properties of the polymer coatings: (**a**) thermogravimetric analysis pyrograms and (**b**) scanning calorimetry thermograms of the C-GCL1, C-GCL5, and C-GCL10.

The poly(urethane acrylate) network coatings containing the self-healing unit were prepared from DABBF, the corresponding GCL polymers, and Desmodur N3300 implementing the conventional drawdown bar coating method. The thermal stabilities of the C-GCL coatings were investigated by TGA (Figure 3a). All C-GCLs were stable up to 180 °C. The values for the glass-transition temperature (*T*_g_) of the C-GCLs were measured by DSC (Figure 3b). The *T*_g_ values of the C-GCLs decreased as the bristle length of the repeating unit increased; notably, increases in the mentioned parameter are associated with decreases in the crosslinking density of the polymer networks.

### 3.2. Results of Nanoindentation Tests Conducted on the C-GCL Coatings

**Figure 4:**
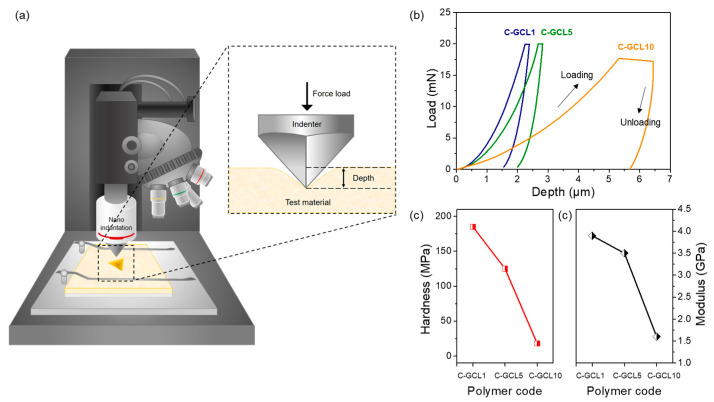
Nanoindentation tests conducted on the C-GCL coatings: (**a**) nanoindentation tester setup, (**b**) load–displacement curves, and (**c**) values for the indentation hardness (*H*_IT_) and indentation modulus (*E*_IT_) of the C-GCL coatings.

In Figure 4 are reported the typical load–displacement curves obtained following implementation of nanoindentation tests on the C-GCL coatings. All C-GCLs exhibited plastic deformation in response to applied normal forces. Moreover, as the bristle length of the polymer increased, the *h*_max_ and *h*_f_ values increased, but the d*P*/d*h* values in the unloading step decreased, which we attributed to the decrease in crosslinking density. The calculated *H*_IT_ and *E*_IT_ values of the C-GCL coatings also decreased as the bristle length of the polymer was increased. Especially, compared to the C-GCL1 and GCL5 coatings, the corresponding C-GCL10 coating exhibited much lower *H*_IT_ and *E*_IT_ values due to its low crosslinking density and glass-transition temperature. Both C-GCL1 and C-GCL5 coatings with high *H*_IT_ and *E*_IT_ values tend to resist deformation against the applied stress. In this case, when scratched, the applied stress will easily be accumulated on the surface and then dissipated by the thermal energy. On the other hand, the surface of the C-GCL10 coating will be deformed to dissipate the applied stress.

### 3.3. Results of the Scratch and Damage-Reporting Tests Conducted on the C-GCL Coatings

**Figure 5:**
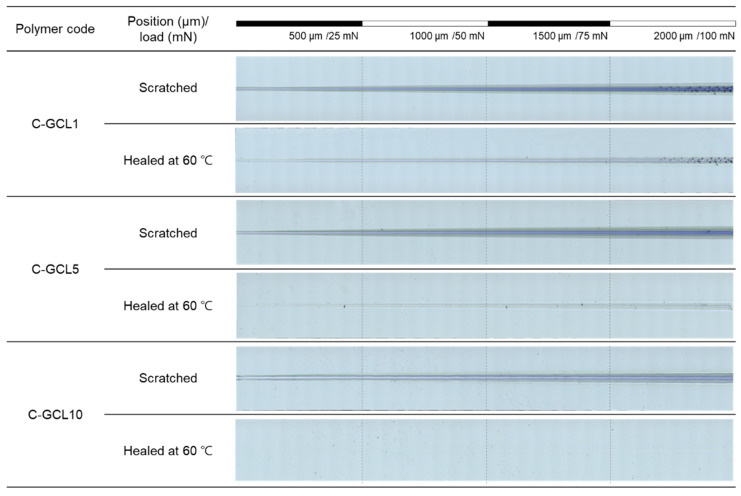
Optical microscopy images of the scratched and healed surfaces of the C-GCL coatings.

The influence of the scratch load on the breakage response of the C-GCL coatings was determined by implementing the scratch test method, whereby a deforming load with a force that increased progressively from 2 to 100 mN was applied (Figure 5).

All C-GCL coatings exhibited plastic deformation at the initial stage of the test (against a small load). As the deforming load increased, however, the mechanophore unit (DABBF) in the C-GCL coatings began to dissociate, dissipating the loading energy.

**Figure 6:**
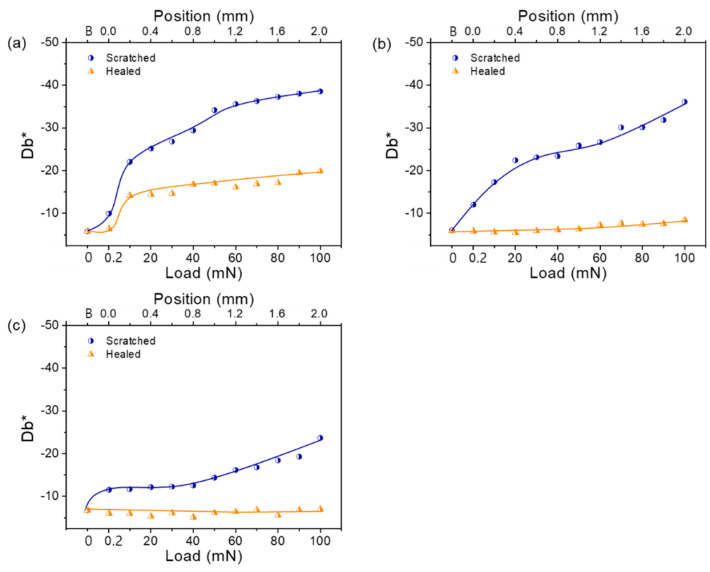
The color transitions (expressed in Db* values) of the damaged and healed surfaces of the C-GCL coatings as a function of the applied loads: (**a**) C-GCL1, (**b**) C-GCL5, and (**c**) C-GCL10.

The color transitions of the scratched C-GCLs, which reflect the degree of dissociation of the DABBF unit, are quantitatively presented in Figure 6 in terms of the various C-GCLs’ Db* values. As the applied force increased, the Db* value drastically decreased, as indicated by the appearance of the scratched position of the C-GCL surfaces, which changed from transparent (colorless) to blue. The Db* values of hard C-GCL coatings decreased more rapidly at smaller deforming loads than the soft C-GCL coatings (C-GCL1 > C-GCL5 > C-GCL10).

In general, hard coatings with high crosslinking density tend to exhibit greater scratch resistance than soft coatings. However, in the present work, the hard C-GCL1 and C-GCL5, which are characterized by high *E*_IT_ and *T*_g_ values (*T*_g_ >> 25 °C), proved to be more vulnerable to surface rupture than the soft C-GCL10 in the rubbery state (*T*_g_ < 25 °C). This interesting observation is attributable to a massive dissociation of the DABBF units, which resulted in the loss of numerous crosslinking points in the hard coating. Indeed, after a massive loss of crosslinking points, the coating is no longer a tough material characterized by high crosslinking density. In addition, C-GCL1 exhibited greater plastic deformation than C-GCL5 and C-GCL10 under the experimental conditions (room temperature). In contrast to this result, evidence from our previous investigations indicated that a thermally reversible dynamic poly(urethane arylate) network with similar chemical structure and material properties to the C-GCL coatings had much higher surface toughness than C-GCL coatings.

Previously, Ostuka et al. investigated the activation and recombination process of DABBF units in the hard and soft domains of a polymer film in response to the application of stretching and grinding stress modes. In their experiments, they observed that the DABBF units in the hard polystyrene domains were not cleaved as a result of the stretching process, but they were activated when the film was ground. In our system, the failure of the DABBF units to elicit a response to the external stimulus in the C-GCL coatings is similar to the result of the grinding stress mode, since two-dimensional stresses (vertical and horizontal stresses) are applied to C-GCL polymers bound to their substrates [25].

### 3.4. Scratch-Healing Performance of the C-GCL Coatings

The scratch-healing performance of the C-GCL coatings was characterized by heating each material at 60 °C for 6 h.

The width-based scratch-healing efficiency (*%WSHE*) was calculated employing Equation (1):

(1) $$\%WSHE=\left[\frac{R_{w,s}-R_{w,h}}{R_{w,s}}\right]\times 100$$

where *R_w_*_,*s*_ and *R_w_*_,*h*_ are the residual width after scratch formation and the residual width after the healing process, respectively.

**Figure 7:**
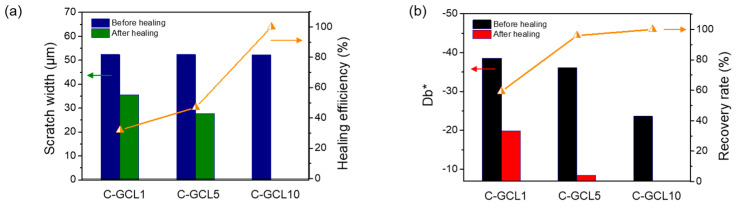
Self-healing and damage-reporting performance of the C-GCL coatings: (**a**) scratch widths and width-based scratch-healing efficiencies of the C-GCL coatings (loading force = 100 mN); (**b**) damage-reporting and diarylbibenzofuranone recombination efficiencies of the C-GCL coatings.

The calculated *%WSHE* values for the C-GCL coatings at a thickness of 2000 μm are presented in Figure 7a. As expected, the self-healing performance of the C-GCL coatings increased as the crosslinking density increased (C-GCL10 >> C-GCL5 > C-GCL1) because of the enhanced polymer-chain mobility. The ruptured surface area of the C-GCL10 coating was also efficiently diminished by the scratch-zipping process (polymer-chain reflow). By contrast, the zipping process of the ruptured C-GCL5 was much more limited than that of C-GCL10, producing the remaining ruptured surface. In the case of C-GCL1, even the remaining scratch formed by plastic deformation as well as the ruptured region was not healed due to the low polymer-chain mobility that characterizes this coating.

We investigated the reconstruction of destroyed crosslinking points in the C-GCL coatings by observing the disappearance of the color due to the DABBF units. As can be evinced from Figure 6 and Figure 7b, the color of the damaged regions of C-GCL10 and C-GCL5 completely disappeared after the healing process, indicating that the recombination of DABBF occurred in high yield. By contrast, the blue color of C-GCL1 did not disappear completely after the scratch-healing process, and it remained in the central position of the scratch path and the massively ruptured area. This result was attributed mainly to C-GCL1 being characterized by higher crosslinking density than C-GCL5 and C-GCL10. In the case of C-GCL5, the blue color of the scratch path and the massively ruptured area after the scratch-healing process disappeared, but the damaged surface did not fully recover its original shape.

The contact of the DABBF units present in the polymer networks with high crosslinking density was highly restricted by the limited polymer-chain movements. Notably, the disappearance of the blue color of the damaged area indicates recombination of the DABBF units. In the case of C-GCL5, under the described healing conditions, the polymer-chain movement is sufficient for the recombination of nearby DABBF units in both deformed and ruptured area to take place, but the said movement is not enough for the polymer to flow over a wide range. Meanwhile, the movements of the polymer chains in C-GCL1 are more highly restricted than those of the polymer chains in C-GCL5 due to C-GCL1′s high crosslinking density. Under the described healing conditions, the DABBF units in the ruptured area (not in the scratch path) of C-GCL1 cannot recombine.

Notably, Otsuka et al. observed similar trends to those reported in the present study. In their system, the recombination of the DABBF units in the rigid silica network was much slower than that observed in the soft poly(butyl acrylate) domain [26].

Based on the results of the aforementioned experiments, we can conclude that, in order to achieve a satisfactory damage-reporting performance, damage-reporting coatings based on dynamic covalent bonds need to be characterized by high *G′* and *T_g_* values (*T*_g_ >> room temperature). However, if the values of the said parameters are too high, the polymer-chain mobility is restricted, resulting in a low recombination yield of the dynamic covalent bond in the polymer. Indeed, high values for *G′* and *T_g_* limit the repeated use of the damage-reporting capability of the polymer coatings. However, if the *G′* value of the coating is too low, the stress required to activate the cleavage of the dynamic covalent bond increases, which means that the sensitivity of the damage-reporting system decreases.

In terms of self-healing, the polymer coatings with low *G′* values exhibit high self-healing performance. This trend is mainly attributed to an increase in polymer-chain mobility. Unlike the conventional self-healing coating system based on dynamic covalent bonding, the surfaces of hard polymers exhibited inferior toughness to those of soft polymers because the crosslinking point of hard coatings is more fragile than that of soft coatings. In addition, ruptures in the surfaces of hard coatings are permanent because recombination of the DABBF units (crosslinking point) only occurs with adjacent DABBF units, which means that polymer-chain interdiffusion in the polymer coating is no longer available.

## 4. Conclusions

In this paper is described a detailed investigation of the influence of the material properties of stress-sensitive dynamic polymer network coatings on their damage-reporting and damage-healing performances. Our observations of the damage-reporting and self-healing process of the dynamic polymer network coatings revealed that material properties such as those identified by the parameters *E*_IT_, *H*_IT_, and *T*_g_, as well as the toughness of the said coatings, greatly influence the self-healing performance of the reversible polymer network coatings containing the DABBF units. In terms of the damage-reporting performance, hard polymer coatings exhibited superior performance to soft polymer coatings because, in the case of hard polymer coatings, the applied stress can mainly be used to break the DABBF units, thus minimizing other energy dissipation modes, such as elastic and plastic deformations. By contrast, hard polymer coatings showed inferior self-healing performance to their soft counterparts because the self-healing process of the intrinsic self-healing polymers was strongly influenced by the polymer-chain interdiffusion effect. These features of the dynamic polymer network coatings are unique; they are not observed in systems like elastomers, films, and hydrogels, where the polymer networks are not bound to the substrate surface. Therefore, we conclude that controlling the polymer’s physical properties is a key factor in designing scratch-healing polymer network coatings based on damage-reporting mechanophores. Notably, in the present system, C-GCL5 exhibited excellent damage-reporting and self-healing performance.

## Figures and Tables

**Table 1:** Molecular weights and molecular-weight distributions of the various oligo(ε-caprolactone) methacrylate monomers (MCLs).

Monomer Code	${\bar{DP_{TV}}\;}^{a}$	${\bar{DP_{CV}}\;}^{b}$	$\bar{M_{n}}\;(g/\mathrm{mol}){\;}^{c}$	$\bar{M_{w}}\;(g/\mathrm{mol}){\;}^{c}$	*Ð*_M_^c^
MCL1	1	1.8	340	450	1.3
MCL5	5	5.5	1080	1310	1.2
MCL10	10	10.3	1500	1710	1.1

^a^$\bar{DP_{TV}}$ and $\bar{DP_{CV}}$ indicate the theoretical and calculated degrees of polymerization, respectively. ^b^ Determined by ^1^H-NMR spectroscopy. ^c^ Determined by size-exclusion chromatography.

**Table 2:** Chemical compositions, molecular weights, and molecular-weight distributions of the hydroxyl-terminated graft copolymers (GCLs).

Polymer Code	MCL ^a^ Contents (mol%) ^b^		$\bar{M_{n}}\;(\mathrm{kg}/\mathrm{mol})$	$\bar{M_{w}}\;(\mathrm{kg}/\mathrm{mol})$	*Ð*_M_^c^
	Theoretical	Calculated			
GCL1	20	20	17.0	25.6	1.5
GCL5	20	21	25.0	32.5	1.3
GCL10	20	21	34.0	44.2	1.3

^a^ MCL: oligo(ε-caprolactone) methacrylate monomer. ^b^ Determined by ^1^H-NMR spectroscopy. ^c^ Determined by size-exclusion chromatography.

## Data Availability

Not applicable.

## Sample Availability

Samples of the compounds are not available.
